# Evaluating the reliability of a microperimetry-based method for assessing visual function in the junctional zone of geographic atrophy lesions

**DOI:** 10.1186/s40942-024-00624-7

**Published:** 2025-01-07

**Authors:** A. Yasin Alibhai, Eric M. Moult, Muhammad Usman Jamil, Khadija Raza, Marco U. Morales, Ramiro Ribeiro, Caroline R. Baumal, James G. Fujimoto, Nadia K. Waheed

**Affiliations:** 1 Boston Image Reading Center, Boston, MA USA; 2 Department of Electrical Engineering and Computer Science, and Research Laboratory of Electronics, Massachusetts Institute of Technology, Cambridge, MA USA; 3 New England Eye Center, Tufts Medical Center, Tufts University School of Medicine, Boston, MA USA; 4 Apellis Pharmaceuticals, Waltham, MA USA

**Keywords:** Microperimetry, Geographic atrophy, Fundus auto fluorescence, Age-related macular degeneration, Scotomatous points

## Abstract

**Purpose:**

To assess the repeatability of a microperimetry methodology for quantifying visual function changes in the junctional zone of eyes with geographic atrophy (GA) in the clinical trial context.

**Methods:**

A post hoc analysis of the OAKS phase III trial was conducted, which enrolled patients with GA secondary to age-related macular degeneration. Microperimetry using a standard 10 − 2 fovea centered grid was performed at baseline and follow-up visits. GA regions were traced on fundus autofluorescence (FAF) images. Two graders independently registered baseline microperimetry images with baseline FAF images in a sampling of 30 eyes from the OAKS study. Agreement between the two graders’ assessments of mean sensitivity and the number of scotomatous points within a ± 250 𝜇m GA junctional zone was assessed.

**Results:**

The intraclass correlation (ICC) and coefficient of repeatability (CoR) for the mean junctional zone sensitivity were 0.987 and 0.214 dB, respectively. The ICC and CoR for the total number of scotomatous points within the junctional zone were 0.991 and 1.42, respectively.

**Conclusions:**

The repeatability of the methodology and its compatibility with standard MP acquisitions appear to make it well-suited for identifying and analyzing retinal sensitivity within high-risk areas of the retina.

**Summary:**

We present a microperimetry-based methodology for assessing visual function changes in the junctional zone of geographic atrophy lesions using a standard 10 − 2 fovea centered grid in a clinical trial context. The approach’s repeatability and compatibility with standard microperimetry grids may make it useful for assessing the effects of GA therapeutics.

**Supplementary Information:**

The online version contains supplementary material available at 10.1186/s40942-024-00624-7.

## Introduction

Geographic atrophy (GA), the late stage of non-exudative age-related macular degeneration, significantly reduces quality of life [1, 2]. The consequences of visual impairment in GA patients include an increased risk of falls, difficulty reading, driving, and recognizing faces, and ultimately, the loss of independence [3, 4]. In 2023, two complement inhibitors were approved by the Food and Drug Administration (FDA), becoming the first approved therapies for treating GA [5, 6]. These therapies were shown to significantly slow the enlargement of GA as measured on fundus autofluorescence (FAF) [7, 8]. However, for these and future GA therapeutics, it is important to understand their effects on measures of visual function in addition to their effects on structural measures.

Sensitive and reliable measurement of visual function in GA patients remains a challenge. The standard assessment of visual acuity is typically performed using eye charts, which is a reliable and reproducible test when patients have a healthy macula and good fixation. In patients with extrafoveal lesions, there can be significant GA growth without any effect on visual acuity, even with patients complaining of worsening visual function. Similarly, once the central fovea is involved, there can be further growth of the GA lesion without additional changes in visual acuity—in this situation, too, the patient’s functional capacity may decline as their scotoma enlarges. Therefore, it is important to develop approaches that can assess the visual function changes that accompany the structural changes that occur as GA lesions enlarge.

Microperimetry (MP) provides a functional mapping of the retina that is precisely correlated to fundus anatomy. Because MP assesses light sensitivity at specific, predefined retinal loci that can be longitudinally tracked, MP may be a sensitive measure of visual function changes in patients with GA [9, 10]. Nevertheless, conventional MP analyses (e.g., mean sensitivity across all stimulus points) are limited in that a substantial proportion of stimuli may fall within the region of atrophy or be located far from the atrophic region, particularly when large sparse grid distribution are selected, and are, therefore, unlikely to change as the GA lesion enlarges. This decoupling may lead to an underestimation of therapeutic effects [11]. Thus, developing and evaluating approaches that analyze MP sensitivities within a lesion-specific junctional zone may lead to more sensitive measurements of the effects that GA therapeutics have on visual function [9, 10]. The aim of this study is to assess the repeatability of an MP methodology for quantifying visual function changes in GA junctional zones in a clinical trial context.

## Methods

### Study design

The repeatability of our MP analysis workflow was evaluated on a cohort of GA patients from the OAKS study (NCT03525600) [7]. The OAKS study was a 24-month, multicenter, randomized, double-masked, sham-controlled, phase 3 study, which enrolled patients at 110 clinical sites. The study adhered to protocols approved by the institutional review board of each site and complied with the Declaration of Helsinki. The inclusion and exclusion criteria of the OAKS study are described elsewhere [8]. Patients were randomly assigned (2:2:1:1), i.e., pegcetacoplan monthly, pegcetacoplan every other month, sham injection monthly, or sham injection every other month, by a central web-based randomization system to intravitreal 15 mg per 0.1 mL.

### MP testing

MP testing was conducted using the Macular Integrity Assessment (MAIA) device (iCare, Padova, Italy) at baseline and every 6 months for up to 24 months. All follow-up MP acquisitions were obtained using a ‘follow-up’ mode to allow registration to the baseline acquisition. MP testing was conducted in a dark room under pharmacologic pupil dilation while the contralateral eye was patched. MP testing was performed using a rectilinear 10 − 2 grid distribution (68 stimulus points; Goldman Size III (0.43º diameter)) centered on the anatomic fovea, with a 4 − 2 staircase threshold strategy, mesopic background luminance of 4 asb (1.27 cd/m^2^), and a 1º diameter red central fixation target. All MP testing was performed prior to any imaging to prevent photoreceptor bleaching.

### Fundus autofluorescence imaging and baseline lesion tracing

Fundus autofluorescence (FAF) imaging was performed using the Spectralis HRA + OCT (Heidelberg Engineering, Heidelberg, Germany) at all study visits. In the high-speed mode, a 30° × 30° field centered on the fovea was imaged. FAF images consisted of 768 × 768 pixels, and average number of sampling frames set between 15 and 25. Based on FAF image tracings made as part of the OAKS Phase 3 GA study, baseline GA tracings for this study were performed by A. Y. A., with the minimum lesion size defined as 0.05 mm [2, 12, 13]. Importantly, all subsequently described analysis used the same baseline GA tracings, meaning that GA tracing was not a source of variability in this study. For analysis, each traced GA focus was represented as polygon, comprising a set 2-D vertices.

### Junctional zone MP analysis

**Fig. 1:**
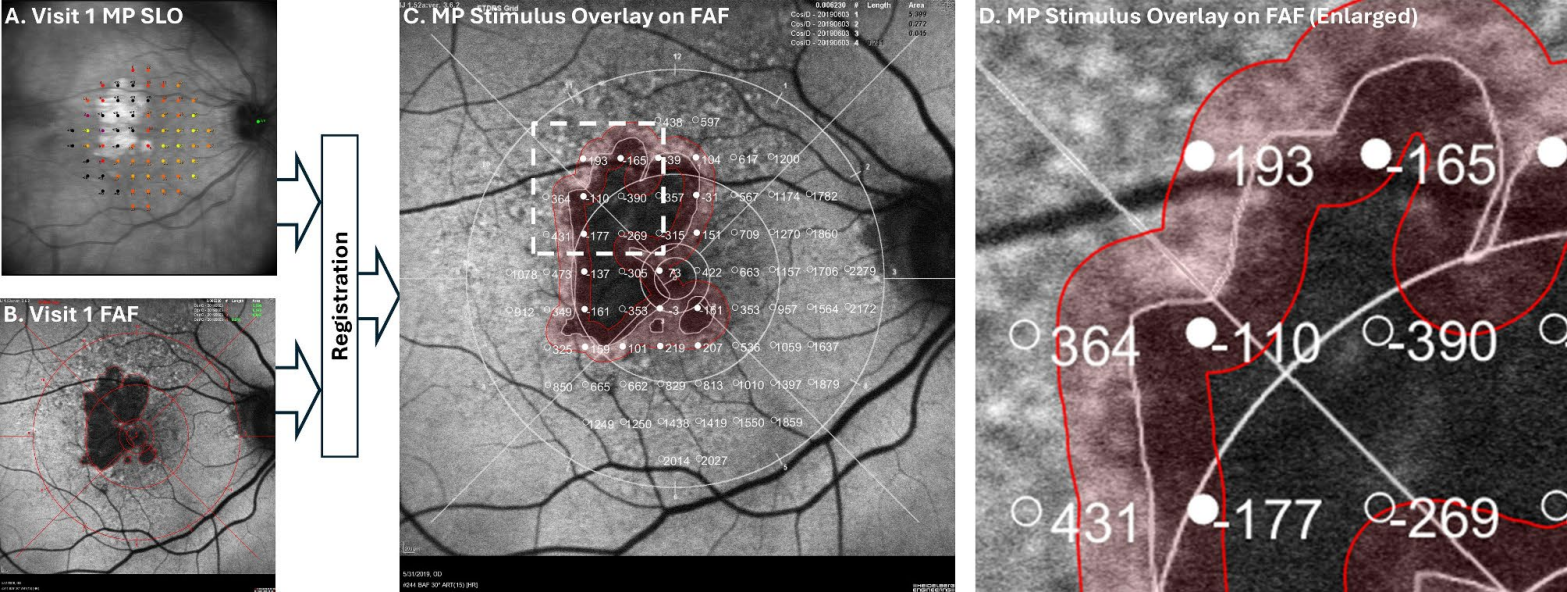
Workflow for longitudinal tracking of microperimetry (MP) sensitivities within the GA junctional zone. Baseline (visit 1) MP scanning laser ophthalmoscopy (SLO) images (panel **A**) are registered with baseline fundus autofluorescence (FAF) images (panel **B**), allowing the MP stimulus points to be overlaid on the FAF image (panels **C, D**). Each MP stimulus point is associated with a signed distance from the lesion margin, as defined by FAF lesion tracing; distances, in micrometers, are shown next to each MP stimulus point. Positive and negative distances correspond to points outside and inside regions of atrophy, respectively. The junctional zone comprising all points within 250 μm of the lesion boundary is shaded red; those MP stimulus positions within the junctional zone have filled markers, and those outside the junctional zone have unfilled markers

The workflow for GA junctional zone MP analysis, which follows the approach used by Hariri [14], is presented in Fig. 1. In brief, using custom MATLAB (MathWorks, Natick, Massachusetts) software, each grader (Grader 1 and Grader 2) registered baseline MP images (sensitivity maps superimposed on their respective scanning laser ophthalmoscopy (SLO) fundus images) to their corresponding baseline FAF images using fiducial markers manually positioned at corresponding bifurcations of the retinal vasculature. For registration, a similarity-type transformation (translation, rotation, and isotropic scaling) was used. This transformation type requires that a minimum of three corresponding points be selected, although the graders were free to select additional points. The estimated transformation was also used to transform the MP stimuli coordinates into the FAF image coordinate frame. For all analysis, MP stimuli were treated as 2-D points, with their coordinates corresponding to the centers of the stimulus. With the GA tracings and MP measurements in the same coordinate frame (i.e., the FAF coordinate frame), the signed Euclidean (i.e., straight-line) distance from each MP stimulus point to the closest point on the baseline GA margin was computed. Negative and positive distances represent stimuli that lie inside and outside the areas of atrophy, respectively. A junctional zone, defined as all fundus positions within 250 μm of the GA margin (including regions both inside and outside the region of atrophy), was automatically generated. The position and width of the junctional zone used in this study matched that used for the post-hoc MP analysis of the OAKS trial [15]. 

### Statistical analysis

Two graders (A. Y. A. and E. M. M.), both experienced with ophthalmic image registration, performed registrations on images from a systematic sampling (every 20th eye) of the OAKS dataset. The repeatability of the mean sensitivity and number of scotomatous points (any stimulus with a -1 dB raw value or < 0 dB on the sensitivity map) within the junctional zone were assessed using Bland–Altman analysis [16], intraclass correlation coefficients (ICCs) and coefficients of repeatability (CoRs), also referred to as the smallest real difference [17]. For Bland-Altman analysis of the mean sensitivity within the junctional zone, individual confidence intervals for the limits of agreement were computed [18]. To assess the repeatability of our MP analysis workflow without considering a particular junctional zone (e.g., ± 250 μm), we considered two measures, both of which incorporate all 68 stimulus points: (1) the repeatability of the signed-distance from each MP stimulus point to the GA margin (“stimulus-to-margin distance”; distances are negative for points within the GA lesion margin and positive for points outside the GA lesion margin), and (2) the distances between the coordinates of corresponding MP stimulus points when transformed by the two readers into the FAF coordinate frame (“stimulus coordinate difference”). The repeatability of the stimulus-to-margin distance was assessed using Bland-Altman analysis, as well as ICC and CoR. Following Taylor et al. [19], linear mixed modeling was used to account for repeated measures. Stimulus coordinate differences were summarized with boxplots and descriptive statistics.

We emphasize that all repeatability analyses in this study assess only the repeatability of the MP *analysis*, which is determined by the repeatability of registering the MP SLO images to the FAF images. In particular, this study did not consider the repeatability of the MP acquisition (both graders used the same MP SLO images), which have been reported by other authors [20], or of the GA lesion tracing (both graders used the same GA lesion tracings).

## Results

**Fig. 2:**
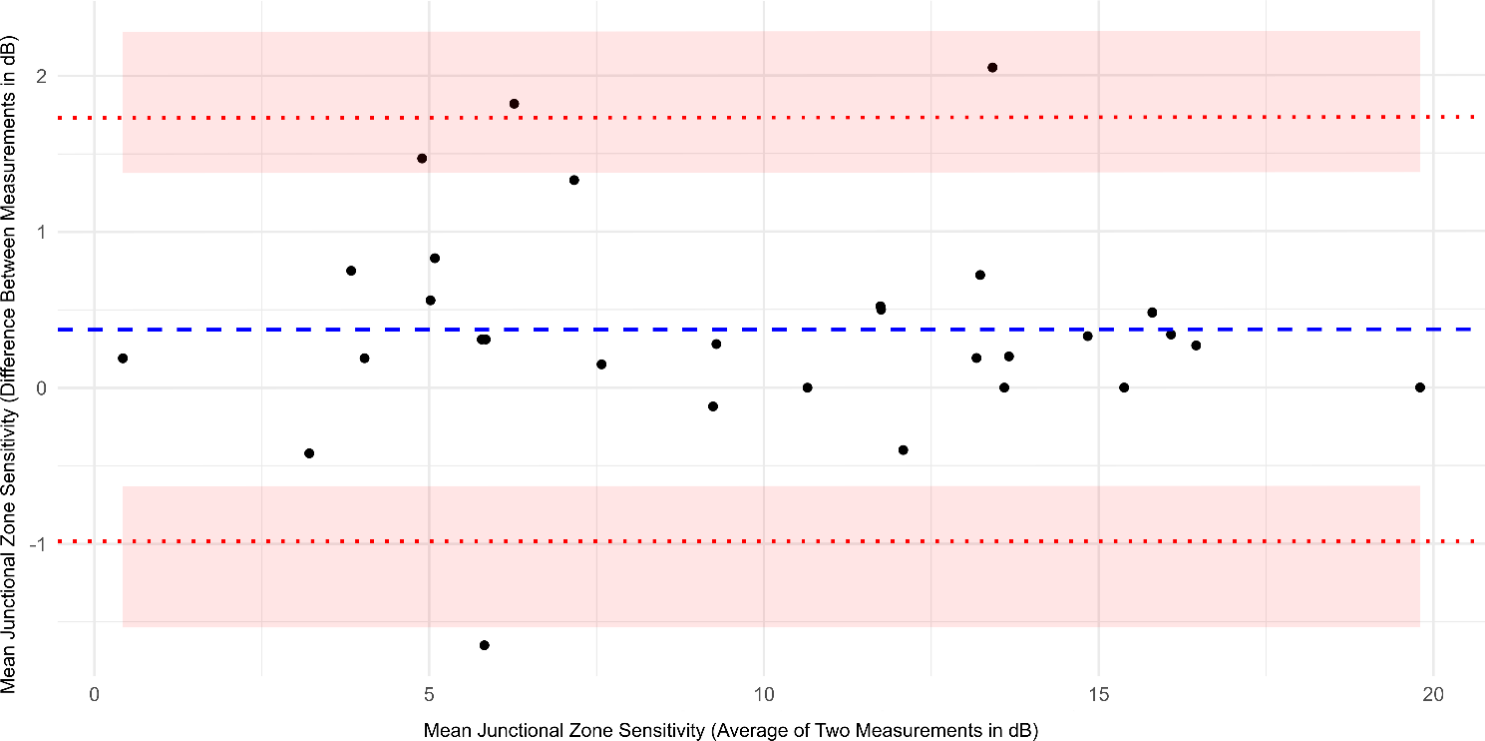
Bland-Altman plot of grader agreement for the mean sensitivity within the junctional zone. Blue dashed line: bias; red dashed lines: upper and lower limits of agreement; red shaded regions: 95% confidence intervals on the limits of agreement; blue markers: eyes

**Fig. 3:**
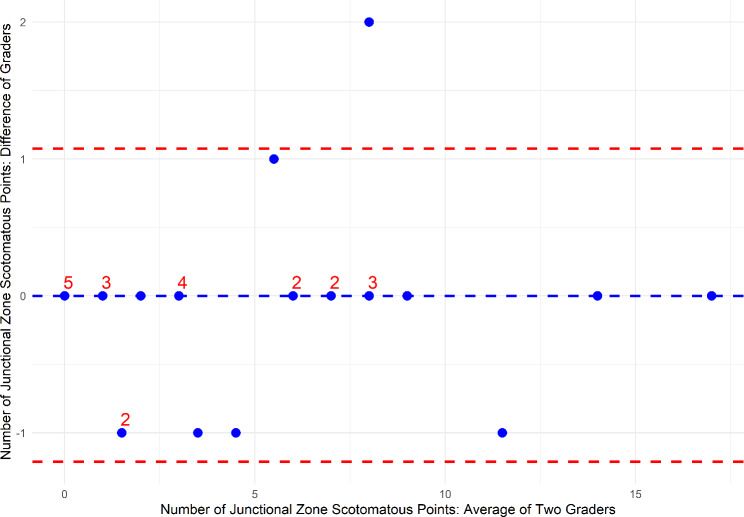
Bland-Altman plot of grader agreement for the total number of scotomatous points within the junctional zone. Blue dashed line: bias; red dashed lines: upper and lower limits of agreement; blue markers: eyes; (To resolve overlapping markers, marker superscripts indicate the number of eyes at that marker)

Images from thirty eyes (24 patients) were registered by two graders. The two graders used an average ± standard deviation of 3.3 ± 0.7 (Grader 1) and 4.6 ± 1.7 (Grader 2) corresponding points to register the MP SLO images to the FAF images. The baseline GA lesions had an average ± standard deviation area of 6.5 ± 3.5 mm [2]. Bland-Altman analysis of the mean sensitivity within the junctional zone showed a bias of 0.37 dB between the two graders, with 90% of the eyes within ± 1.96 SD [95% limits of agreement (LOA): -0.98 dB (upper 95% CI: -0.63 dB, lower 95% CI: -1.54 dB) to 1.73 dB (upper 95% CI: 2.28 dB, lower 95% CI: 1.38 dB); Fig. 2). Bland-Altman analysis of total number of scotomatous points within the junctional zone showed a bias of 0.06 between the two graders, with 96% of the points within ± 1.96 SD (95% limits of agreement (LOA): -1.21 to 1.07; Fig. 3). The ICC and CoR for the mean junctional zone sensitivity were 0.987 and 0.214 dB, respectively. The ICC and CoR for the total number of scotomatous points within the junctional zone were 0.991 and 1.42, respectively.

**Fig. 4:**
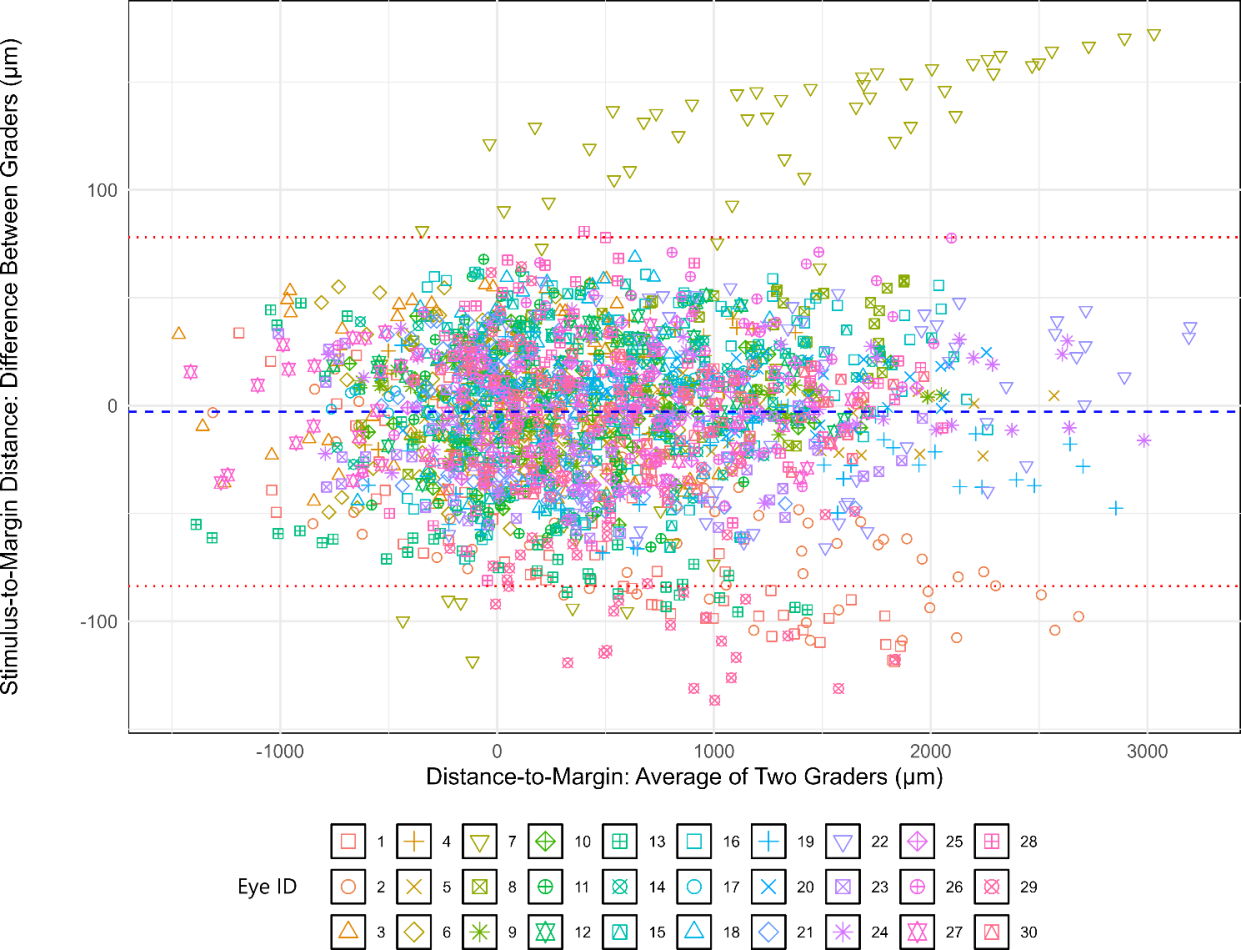
Bland-Altman plot of grader agreement of stimulus-to-margin distances across all measured stimulus points (68 per eye). Blue dashed line: bias; red dashed lines: upper and lower limits of agreement; markers: individual MP stimulus points (MP stimulus points from different eyes have different marker-color combinations)

**Fig. 5:**
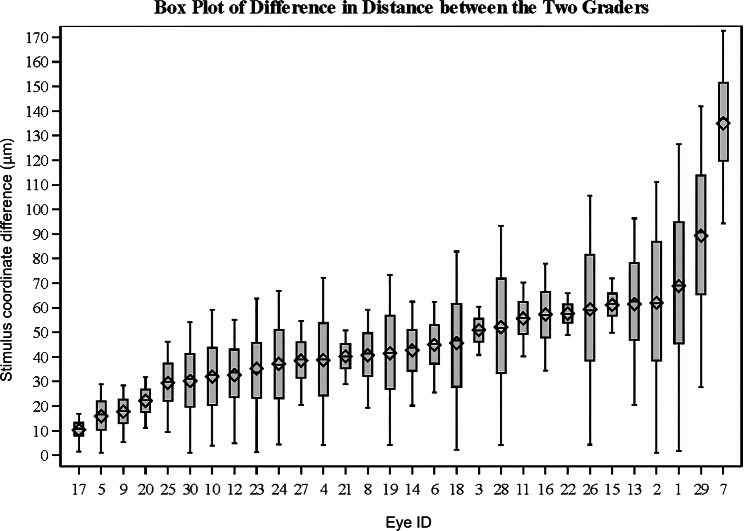
Box plot of the mean and standard deviation of the average stimulus coordinate differences over the 68 MP stimulus points for each eye

**Fig. 6:**
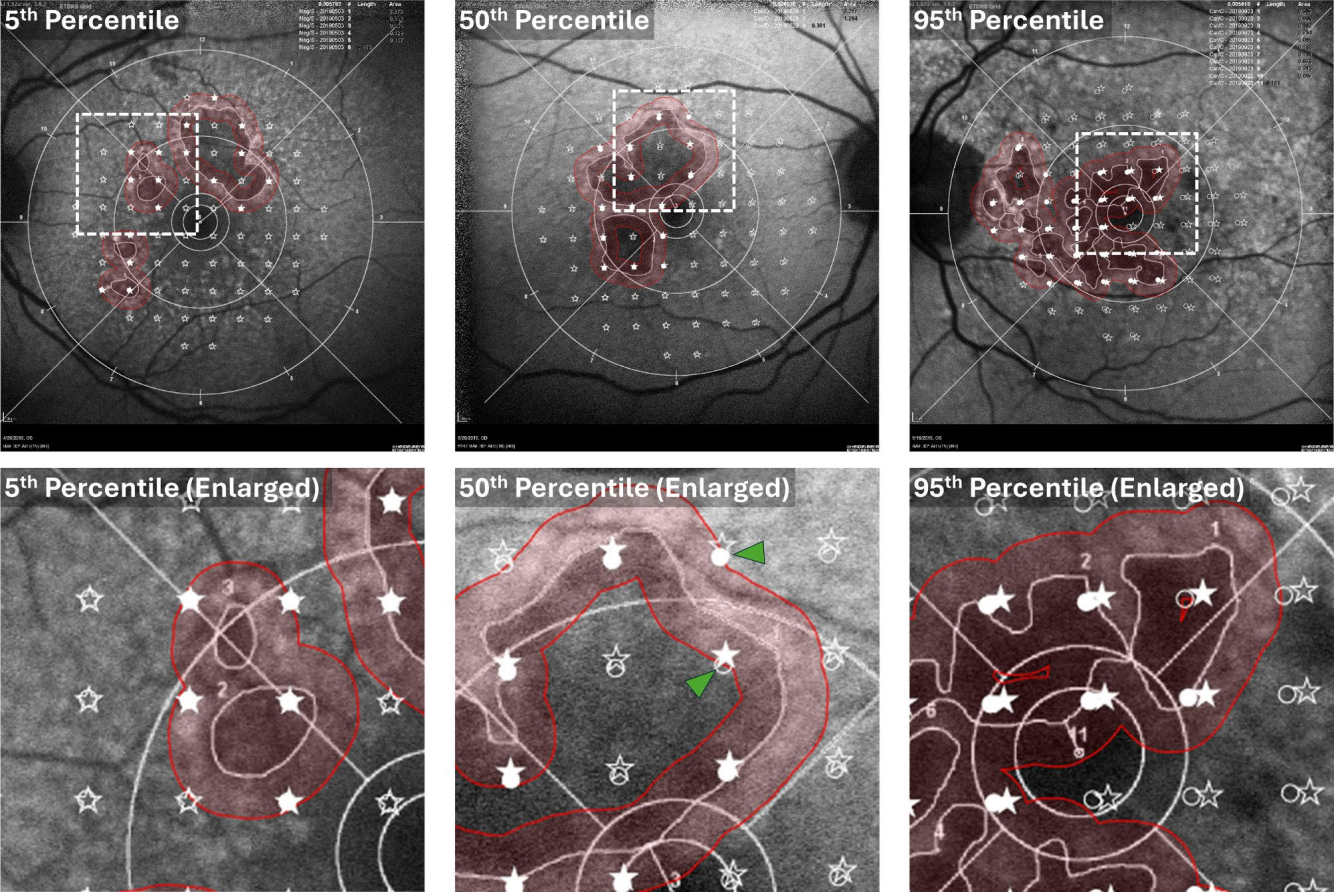
Representative Grader 1 and Grader 2 overlays of the microperimetry (MP) stimulus points on the fundus autofluorescence (FAF) images used for GA tracing. MP stimulus points are indicated by circle markers (Grader 1) and star markers (Grader 2). Filled and unfilled markers correspond to MP points inside and outside of the junctional zone, indicated by red shading, respectively. The images correspond to subjects having the 5th percentile (Subject 5), 50th percentile (subject 19), and 95th percentile (Subject 29) mean stimulus coordinate differences (see Fig. 5). Enlargements of regions of interest specified by dashed boxes (top row) are shown in the bottom row. The green arrowheads indicate grader discrepancies in the assignment of points as being inside or outside of the junctional zone

Bland-Altman analysis of the stimulus-to-margin distance, measured across all stimulus points (68 per eye), showed a mean shift of -2.83 μm between the two graders, with 94% of the points within ± 1.96 SD from the mean shift (95% limits of agreement (LOA): -83.69 μm to 78.03 μm; Fig. 4). Two eyes had larger variabilities (SD of difference > 50 μm) between the two graders, and six were outside the LOA bounds. The ICC and CoR of the stimulus-to-margin distances between the two graders were 0.998 and 102.96 μm, respectively. The stimulus coordinate differences are summarized in Fig. 5. The mean stimulus coordinate difference taken across stimulus points for all subjects was 46.87 ± 23.80 μm. Representative images of the registered MP in the FAF coordinate frame are shown in Fig. 6.

## Discussion

In the present study, we evaluated the repeatability of an MP analysis workflow that is compatible with clinical trial data (10 − 2 MP stimulus grid distribution and FAF imaging for GA tracing) and allows visual function to be assessed in retinal areas that are most likely to be affected by GA growth—namely, the junctional zone comprised of the regions immediately surrounding the GA lesion margin. The repeatability of the method in the context of the mean junctional zone sensitivity and the number of scotomatous points within the junctional zone was excellent. Assessments of stimulus-to-margin distances and stimulus coordinate difference showed reader differences that were generally small compared to the junctional zone width, although there were some outlier cases (Figs. 4 and 5). As the OAKS trial prespecified a secondary outcome measure assessing MP sensitivity within a perilesional zone extending from the lesion margin to 500 μm beyond the lesion, we performed an additional repeatability analysis of our methodology using this perilesional zone. These results, provided in Additional file 1, are largely similar to those from our analysis of the ± 250 μm perilesional zone.

From a subjective review of grader registrations, we suspect that the dominant contribute inter-grader discrepancies is likely the similarity-type image transformation not fully capturing the true deformation between the MP SLO and FAF images. Thus, to reduce inter-reader discrepancies in future studies, more general transformation types that better approximate the true deformation could be used. However, more general transformation types require a greater number of corresponding vessel bifurcations to be selected—in addition to increasing the grading time, selecting more vessel bifurcations can itself be error prone for images with less pronounced vasculature, and may even lead to larger discrepancies.

As in Hariri et al., [14] the MP workflow used in this study analyzes junctional zone sensitivities by transforming MP stimulus coordinates into the coordinate frame used for lesion tracing. After this transformation, the position of each MP stimulus point can be directly related to the GA lesion margin. An alternative approach to analyzing MP sensitivities in the junctional zone, proposed by Meleth et al. and recently used in the post hoc analysis of the Spectri and Chroma lampalizumab trials [21], is to define the junctional zone using the set of scotomatous MP points. Such an approach has the advantage of simplicity (e.g., no image registration required). Furthermore, it can be performed using only MP data, making it particularly well suited to analyses in which GA tracing data are unavailable. However, for standard 10 − 2 MP stimulus grids, the 2-degree stimulus spacing suggests that a junctional zone derived using MP only is likely to be less accurate than a junctional zone derived directly from the registered GA tracing data.

Another approach to measuring junctional zone MP sensitivities is to use patient-tailored MP grids wherein the stimuli are distributed around the lesion margin [10, 22]. An advantage of patient-tailored approaches is that MP measurements are not collected at fundus positions that are decoupled from lesion growth (e.g., regions of atrophy at baseline). Moreover, patient-tailored MP allows stimuli points to be distributed around lesions more uniformly and at higher-density within the junctional zone. A disadvantage is the requirement of customized, lesion-specific grids, which may complicate or be incompatible with current MP workflows and may become complex for certain lesion geometries (e.g., multifocal lesions). Moreover, the optimal grid parameters (e.g., stimulus density and junctional zone dimensions) are not a priori obvious. Indeed, one possible application of the MP approach used in the present study is in helping to design MP grid patterns for future studies using patient-specific MP.

An important limitation of our approach, particularly as applied to standard MP grids, is that the MP stimuli are relatively sparse and are randomly distributed relative to regions of atrophy. In addition to the possibility of missing smaller regions of functional impairment, there is a sparse and unequal sampling of the junctional zones, which we expect to increase variances when estimating treatment effects. One potential mitigation strategy is to model, or otherwise adjust for, the spatial distribution of stimulus points within the junctional zone. An alternate approach is to re-sample the MP measurements (e.g., via interpolation [23]) such that they uniformly tile the junctional zone. While these approaches also have limitations, we hope to explore these approaches in future studies.

## Conclusion

In this paper, we evaluate a microperimetry-based approach for assessing visual function changes in the GA junctional zone in a clinical trial context. The repeatability of the approach and its compatibility with standard MP acquisitions appear to make it well-suited to assessing the effects of GA therapeutics on visual function.

## Electronic supplementary material

Below is the link to the electronic supplementary material.

Supplementary Material 1

Supplementary Material 2

Supplementary Material 3

Supplementary Material 4

## Data Availability

No datasets were generated or analysed during the current study.

## References

[1] Patel PJ, Ziemssen F, Ng E, et al. Burden of illness in Geographic Atrophy: a study of Vision-Related Quality of Life and Health Care Resource Use. Clin Ophthalmol. 2020;14:15. 10.2147/OPTH.S226425. 32021065 10.2147/OPTH.S226425 PMC6955611

[2] Singh RP, Patel SS, Nielsen JS, Schmier JK, Rajput Y, Patient-. Caregiver-, and Eye Care Professional-reported Burden of Geographic Atrophy secondary to age-related Macular Degeneration. Am J Ophthalmic Clin Trials. Published online 2019. https://api.semanticscholar.org/CorpusID:132212717

[3] Fleckenstein M, Mitchell P, Freund KB, et al. The progression of Geographic Atrophy secondary to age-related Macular Degeneration. Ophthalmology. 2018;125(3):369–90. 10.1016/J.OPHTHA.2017.08.038. 29110945 10.1016/j.ophtha.2017.08.038

[4] Kim A, Bansal A, Devine B. Characterizing the Healthcare Resource Utilization and Costs By Disease Severity Among Patients with Geographic Atrophy Secondary to Age-Related Macular Degeneration. Published online 2019. Accessed November 19, 2023. https://digital.lib.washington.edu:443/researchworks/handle/1773/44687

[5] SYFOVRE^®^ (pegcetacoplan injection). Accessed November 19. 2023. https://syfovre.com/.

[6] Iveric Bio Receives U.S. FDA Approval for IZERVAY™ (avacincaptad pegol intravitreal solution), a New Treatment for Geographic Atrophy - Aug 4, 2023. Accessed December 9. 2024. https://newsroom.astellas.us/2023-08-04-Iveric-Bio-Receives-U-S-FDA-Approval-for-IZERVAY-TM-avacincaptad-pegol-intravitreal-solution-,-a-New-Treatment-for-Geographic-Atrophy

[7] Heier JS, Lad EM, Holz FG, et al. Pegcetacoplan for the treatment of geographic atrophy secondary to age-related macular degeneration (OAKS and DERBY): two multicentre, randomised, double-masked, sham-controlled, phase 3 trials. Lancet. 2023;402(10411):1434–48. 10.1016/S0140-6736(23)01520-9. 37865470 10.1016/S0140-6736(23)01520-9

[8] Khanani AM, Patel SS, Staurenghi G, et al. Efficacy and safety of avacincaptad pegol in patients with geographic atrophy (GATHER2): 12-month results from a randomised, double-masked, phase 3 trial. Lancet. 2023;402(10411):1449–58. 10.1016/S0140-6736(23)01583-0. 37696275 10.1016/S0140-6736(23)01583-0

[9] Csaky KG, Patel PJ, Sepah YJ, et al. Microperimetry for geographic atrophy secondary to age-related macular degeneration. Surv Ophthalmol. 2019;64(3):353–64. 10.1016/J.SURVOPHTHAL.2019.01.014. 30703401 10.1016/j.survophthal.2019.01.014 PMC6532786

[10] Pfau M, Jolly JK, Wu Z, et al. Fundus-controlled perimetry (microperimetry): application as outcome measure in clinical trials. Prog Retin Eye Res. 2021;82:100907. 10.1016/J.PRETEYERES.2020.100907. 33022378 10.1016/j.preteyeres.2020.100907 PMC12872260

[11] Markowitz SN, Reyes SV. Microperimetry and clinical practice: an evidence-based review. Can J Ophthalmol. 2013;48(5):350–7. 10.1016/J.JCJO.2012.03.004. 24093179 10.1016/j.jcjo.2012.03.004

[12] Schmitz-Valckenberg S, Brinkmann CK, Alten F, et al. Semiautomated image processing method for identification and quantification of geographic atrophy in age-related macular degeneration. Invest Ophthalmol Vis Sci. 2011;52(10):7640–6. 10.1167/IOVS.11-7457. 21873669 10.1167/iovs.11-7457

[13] Schmitz-Valckenberg S, Sahel JA, Danis R, et al. Natural History of Geographic Atrophy Progression Secondary to Age-Related Macular Degeneration (Geographic Atrophy Progression Study). Ophthalmology. 2016;123(2):361–8. 10.1016/J.OPHTHA.2015.09.036. 26545317 10.1016/j.ophtha.2015.09.036

[14] Hariri AH, Tepelus TC, Akil H, Nittala MG, Sadda SR. Retinal sensitivity at the junctional zone of eyes with Geographic Atrophy due to age-related Macular Degeneration. Am J Ophthalmol. 2016;168:122–8. 10.1016/J.AJO.2016.05.007. 27189929 10.1016/j.ajo.2016.05.007

[15] Chakravarty U, Schwartz R, Guymer R et al. Visual function preservation in patients with geographic atrophy treated with pegcetacoplan: microperimetry analysis from the phase 3 oaks trial. Submitted to Ophthalmology Retina.

[16] Hanneman SK. Design, analysis, and interpretation of method-comparison studies. AACN Adv Crit Care. 2008;19(2):223–34. 10.1097/01.AACN.0000318125.41512.A3. 18560291 10.1097/01.AACN.0000318125.41512.a3 PMC2944826

[17] Vaz S, Falkmer T, Passmore AE, Parsons R, Andreou P. The case for using the repeatability coefficient when calculating test–retest reliability. PLoS ONE. 2013;8(9):e73990. 10.1371/JOURNAL.PONE.0073990. 24040139 10.1371/journal.pone.0073990 PMC3767825

[18] Carkeet A. Exact parametric confidence intervals for bland-Altman limits of agreement. Optom Vis Sci. 2015;92(3):e71–80. 10.1097. 25650900 10.1097/OPX.0000000000000513

[19] Taylor LJ, Josan AS, Jolly JK, Maclaren RE. Microperimetry as an Outcome measure in RPGR-associated Retinitis Pigmentosa clinical trials. Transl Vis Sci Technol. 2023;12(6):4–4. 10.1167/TVST.12.6.4. 37294702 10.1167/tvst.12.6.4 PMC10259674

[20] Higgins BE, Montesano G, Dunbar HMP, et al. Test-retest variability and discriminatory power of measurements from Microperimetry and Dark Adaptation Assessment in People with Intermediate Age-Related Macular Degeneration - A MACUSTAR Study Report. Transl Vis Sci Technol. 2023;12(7). 10.1167/TVST.12.7.19. 10.1167/tvst.12.7.19 PMC10365139 37477933

[21] Chang DS, Callaway NF, Steffen V, et al. Macular Sensitivity endpoints in Geographic Atrophy: exploratory analysis of Chroma and Spectri Clinical trials. Ophthalmol Sci. 2024;4(1):100351. 10.1016/J.XOPS.2023.100351. 37869030 10.1016/j.xops.2023.100351 PMC10587617

[22] Schmitz-Valckenberg S, Bültmann S, Dreyhaupt J, Bindewald A, Holz FG, Rohrschneider K. Fundus Autofluorescence and Fundus Perimetry in the Junctional Zone of Geographic Atrophy in patients with age-related Macular Degeneration. Invest Ophthalmol Vis Sci. 2004;45(12):4470–6. 10.1167/IOVS.03-1311. 15557456 10.1167/iovs.03-1311

[23] Denniss J, Astle AT. Spatial interpolation enables normative data comparison in Gaze-Contingent Microperimetry. Invest Ophthalmol Vis Sci. 2016;57(13):5449–56. 10.1167/IOVS.16-20222. 27760271 10.1167/iovs.16-20222

