# Supplementary material for: Evaluating the reliability of a microperimetry-based method for assessing visual function in the junctional zone of geographic atrophy lesions
Source: Int J Retina Vitreous. 2025 Jan 7;11:1. doi: 10.1186/s40942-024-00624-7 (PMC11707945; doi:10.1186/s40942-024-00624-7)
Supplement: Supplementary file 1 — Supplementary Material 1 [file 40942_2024_624_MOESM1_ESM.docx]

**Additional File 1.**

**0 - 500 µm Junctional Zone Analysis**

Bland-Altman analysis of the mean sensitivity within the junctional zone, defined here as the region between 0 µm and 500 µm beyond the GA margin, showed a bias of 0.06 dB between the two graders, with 96.66% of the eyes within ±1.96 SD [95% limit of agreement (LOA): -1.13 dB (upper 95% CI: -0.82 dB, lower 95% CI: -1.62 dB) to 1.26 dB (upper 95% CI: 1.75 dB, lower 95% CI: 0.95 dB); Additional File 1, Figure 1]. Bland-Altman analysis of total number of scotomatous points within the junctional zone showed a bias of 0.033 between the two graders, with 83.33% of the points within ±1.96 SD (95% limit of agreement (LOA): -0.77 to 0.84; Additional File 1, Figure 2). The ICC and CoR for the mean junctional zone sensitivity were 0.992 and 0.151 dB, respectively. The ICC and CoR for the total number of scotomatous points within the junctional zone were 0.975 and 0.81 dB, respectively.


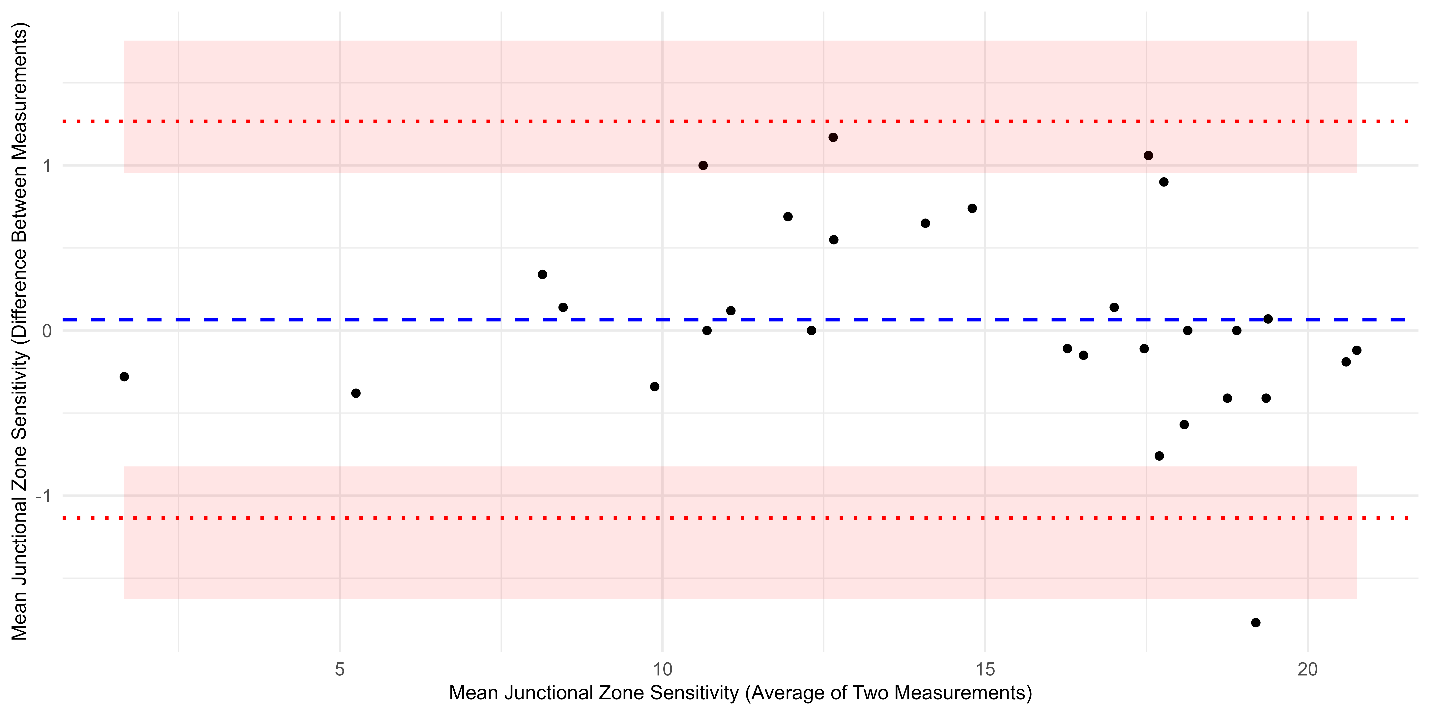


**Additional File 1, Figure 1.** Bland-Altman plot of grader agreement for the mean sensitivity within the junctional zone (0-500 µm). Blue dashed line: bias; red dashed lines: upper and lower limits of agreement; red shaded regions: 95% confidence intervals on the limits of agreement; black markers: eyes.


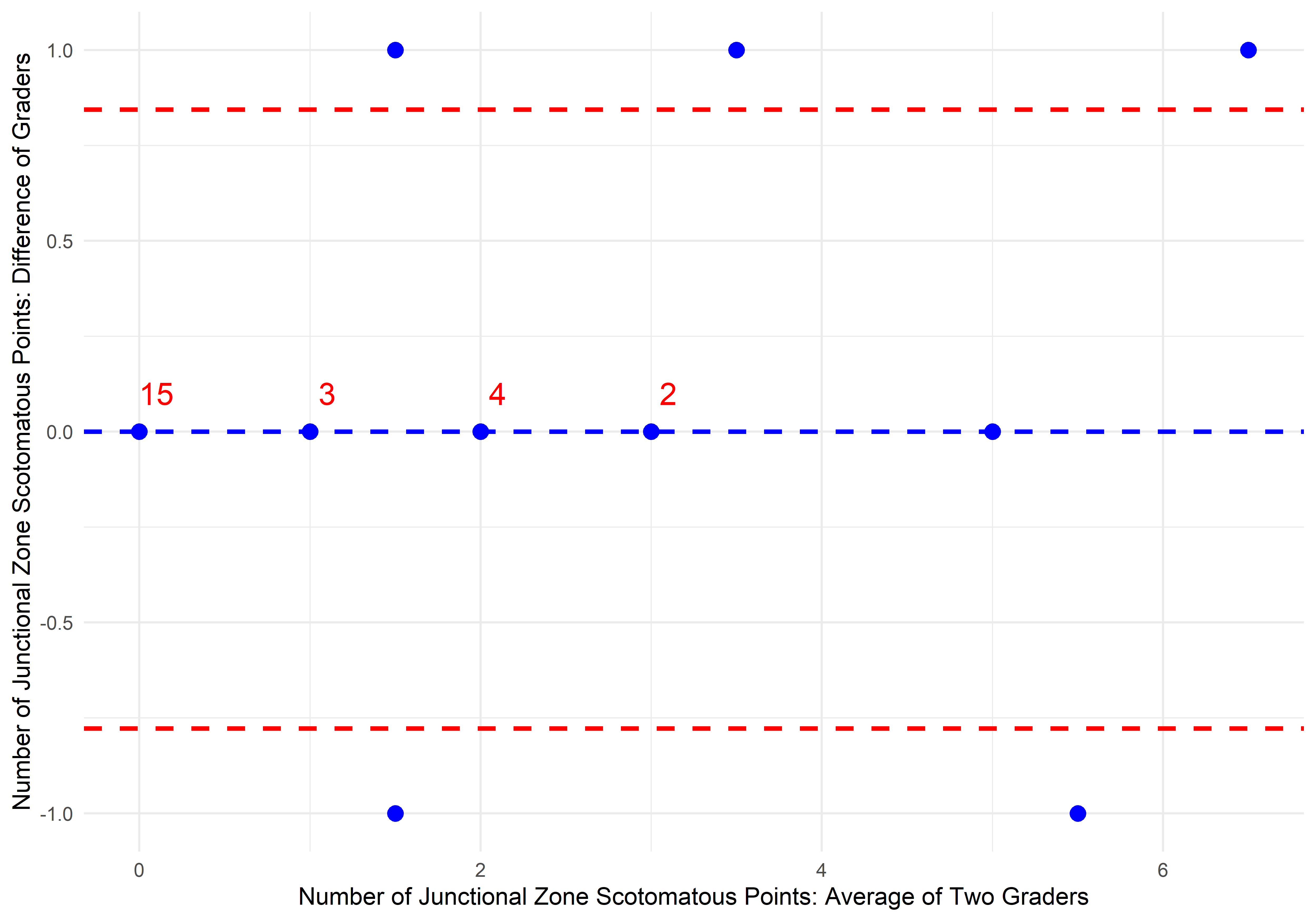


**Additional File 1, Figure 2.** Bland-Altman plot of grader agreement for the total number of scotomatous points within the junctional zone (0-500 µm). Blue dashed line: bias; red dashed lines: upper and lower limits of agreement; blue markers: eyes; (To resolve overlapping markers, marker superscripts indicate the number of eyes at that marker).
