# Supplementary material for: Evaluating the reliability of a microperimetry-based method for assessing visual function in the junctional zone of geographic atrophy lesions
Source: Int J Retina Vitreous. 2025 Jan 7;11:1. doi: 10.1186/s40942-024-00624-7 (PMC11707945; doi:10.1186/s40942-024-00624-7)
Supplement: Supplementary file 2 — Supplementary Material 2 [file 40942_2024_624_MOESM2_ESM.docx]

**Additional File 2.**


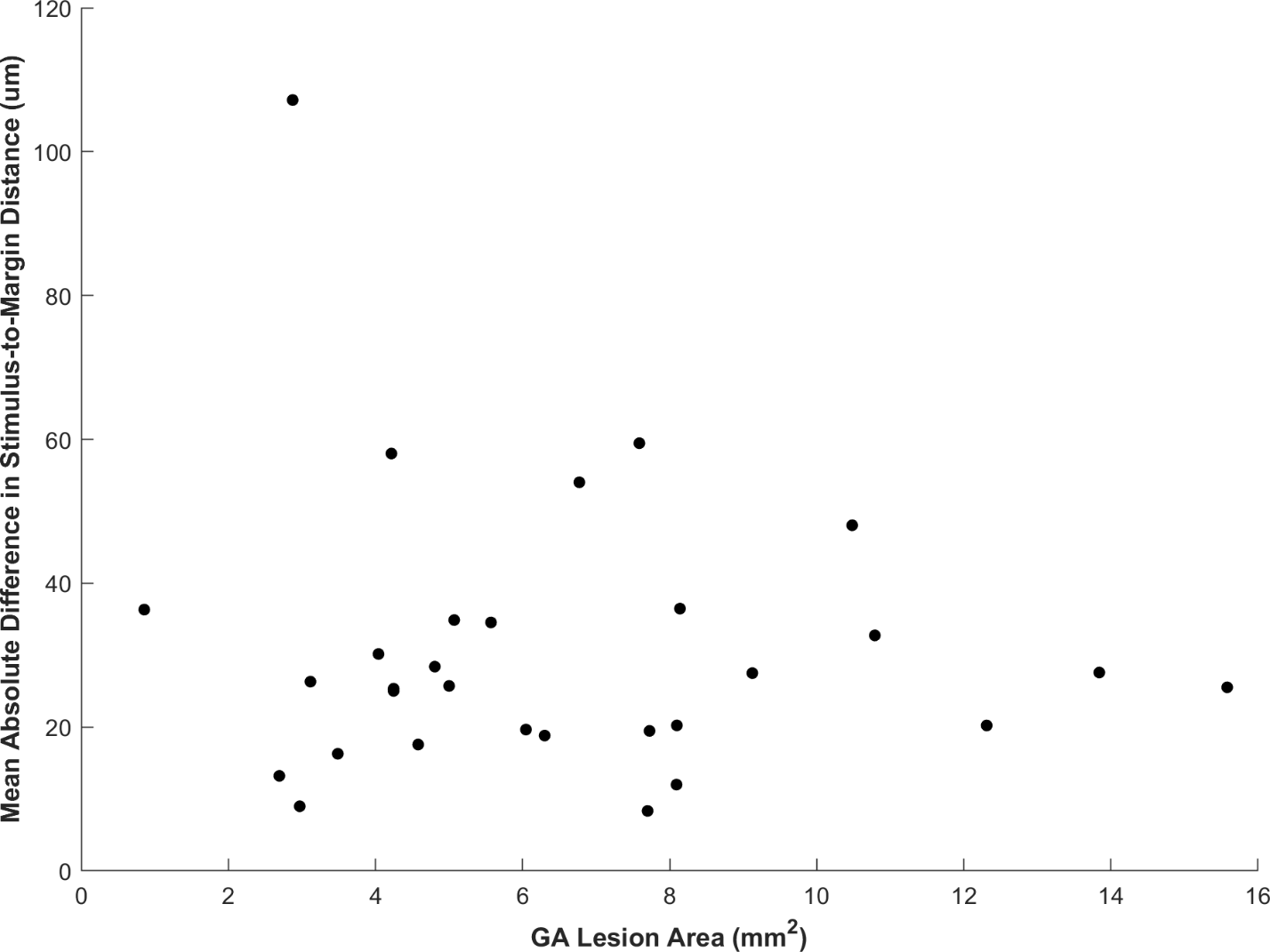


**Additional File 2, Fig. 1.** Scatterplot of the mean absolute difference in stimulus-to-margin distance versus GA lesion area. Each marker corresponds to a single eye. The mean is taken across all 68 stimulus points for each eye.


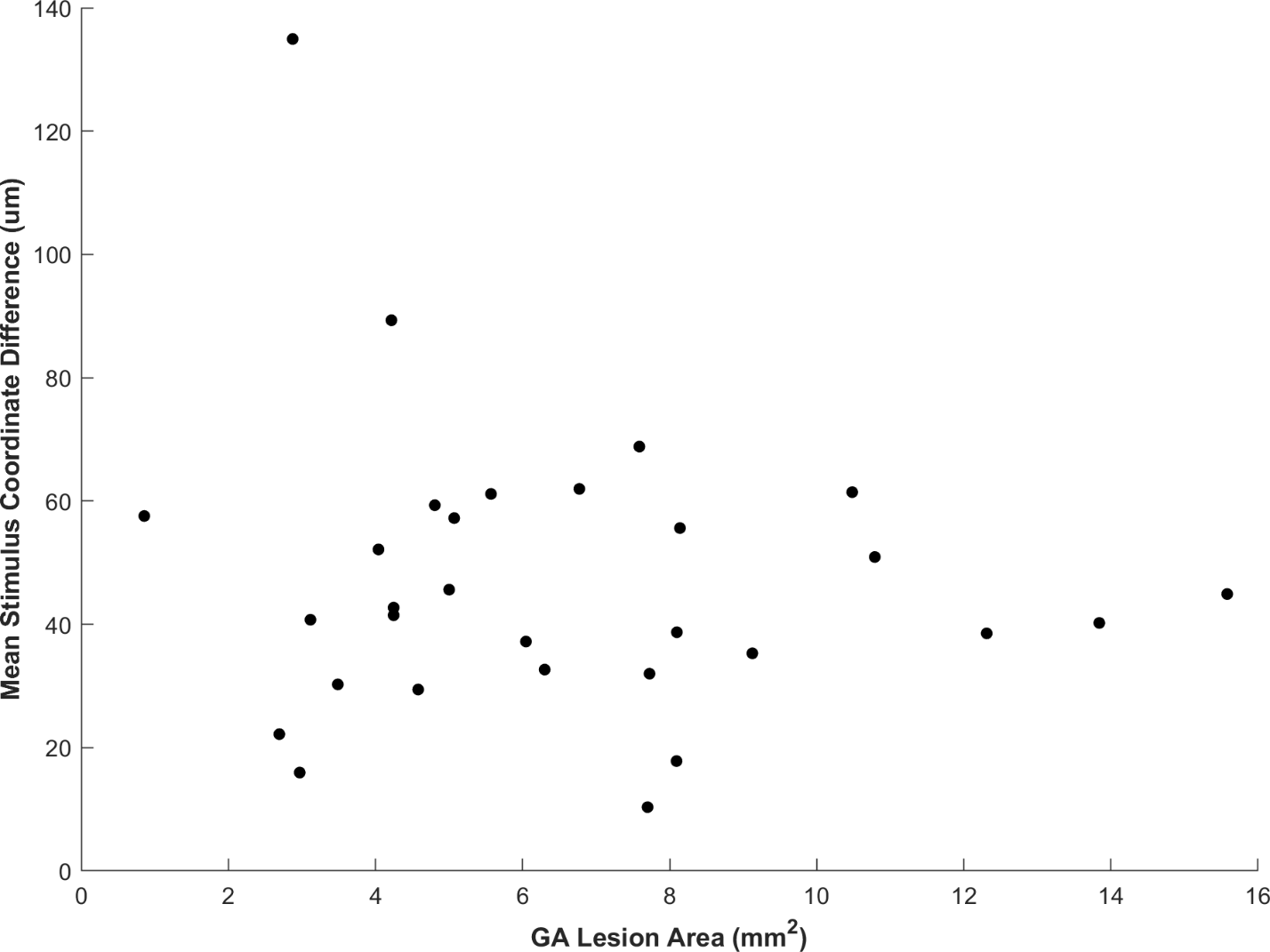


**Additional File 2, Fig. 2.** Scatterplot of the mean stimulus coordinate difference versus GA lesion area. Each marker corresponds to a single eye. The mean is taken across all 68 stimulus points for each eye.
