# Supplementary figures and images for: Evaluating the reliability of a microperimetry-based method for assessing visual function in the junctional zone of geographic atrophy lesions
Source: Int J Retina Vitreous. 2025 Jan 7;11:1. doi: 10.1186/s40942-024-00624-7 (PMC11707945; doi:10.1186/s40942-024-00624-7)

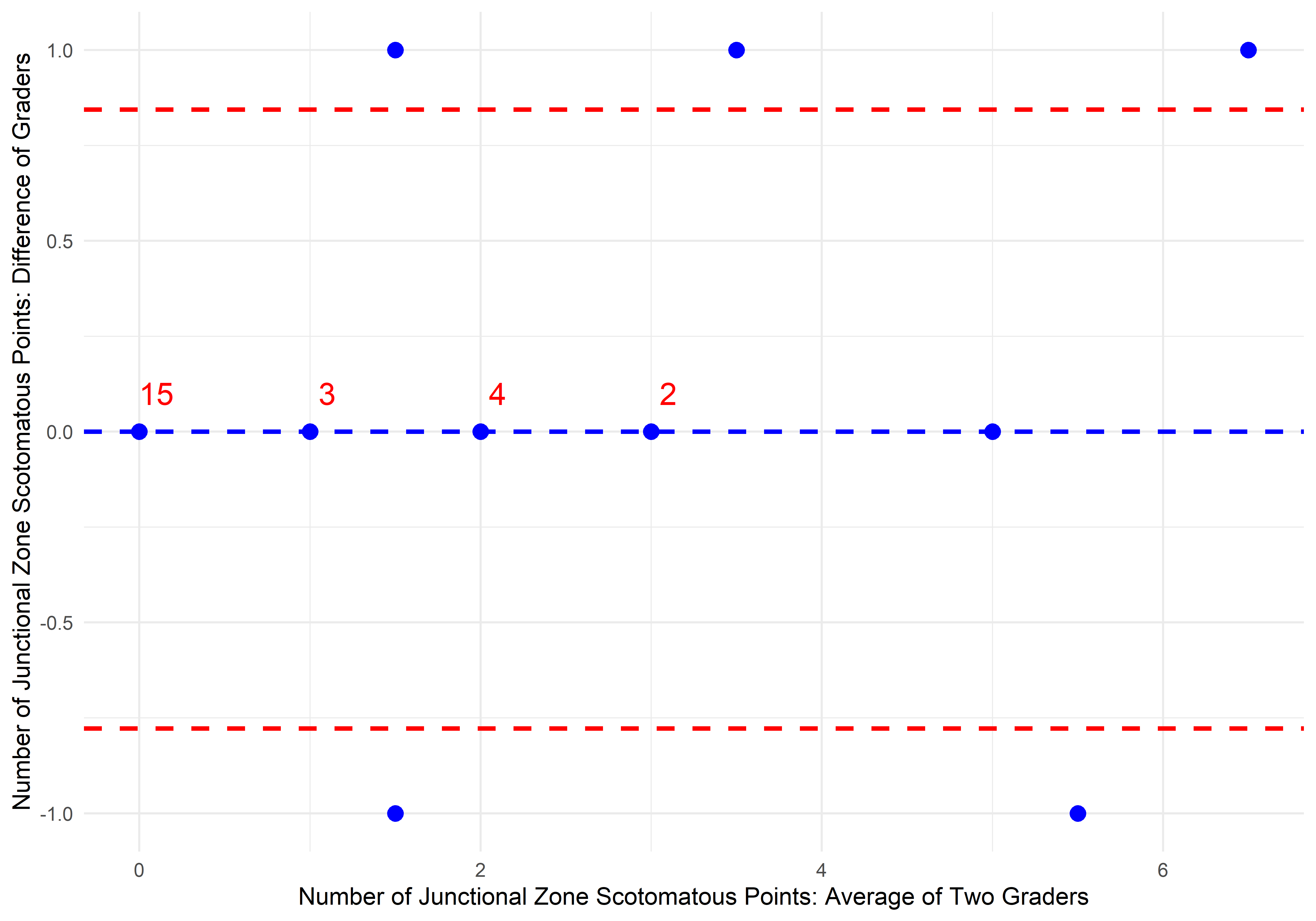

Supplement: Supplementary file 3 — Supplementary Material 3 [file 40942_2024_624_MOESM3_ESM.tif]

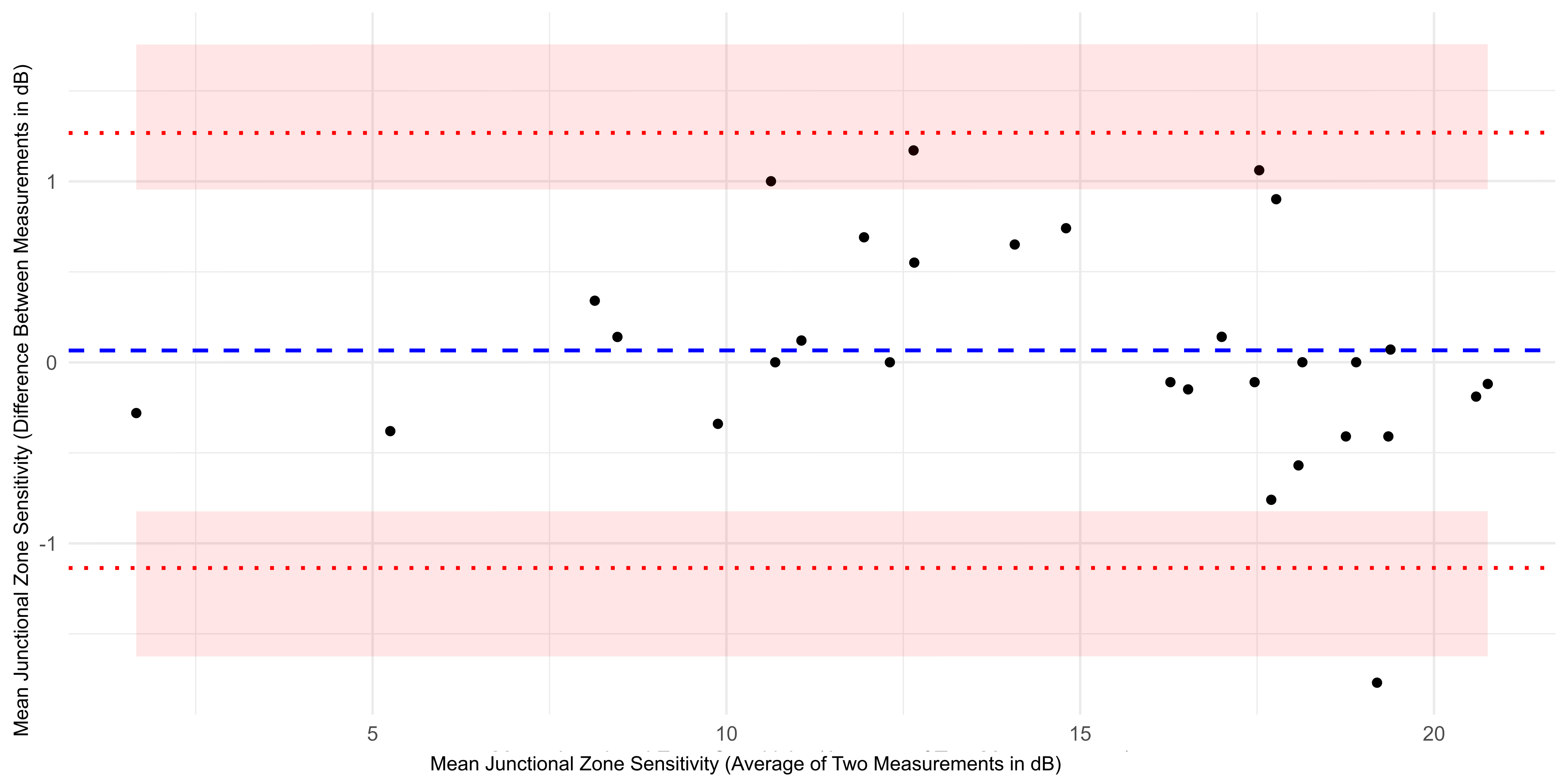

Supplement: Supplementary file 4 — Supplementary Material 4 [file 40942_2024_624_MOESM4_ESM.tif]
